# The Implementation of Dementia Care Mapping in a Randomized Controlled Trial in Long-Term Care: Results of a Process Evaluation

**DOI:** 10.1177/1533317519845725

**Published:** 2019-05-05

**Authors:** Claire A. Surr, Alys W. Griffiths, Rachael Kelley, Ivana Holloway, Rebecca E. A. Walwyn, Adam Martin, Joanne McDermid, Lynn Chenoweth, Amanda J. Farrin

**Affiliations:** 1 Centre for Dementia Research, Leeds Beckett University, Leeds, United Kingdom; 2 Clinical Trials Research Unit, University of Leeds, Leeds, United Kingdom; 3 Leeds Institute of Health Sciences, University of Leeds, Leeds, United Kingdom; 4 Wolfson Centre for Age Related Diseases, Kings College London, London, United Kingdom; 5 Centre for Healthy Brain Ageing, University of New South Wales, Sydney, New South Wales, Australia

**Keywords:** process evaluation, intervention fidelity, psychosocial interventions, care homes, dementia

## Abstract

This study explored intervention implementation within a pragmatic, cluster randomized controlled trial of Dementia Care Mapping™ (DCM) in UK care homes. DCM is a practice development tool comprised of a 5 component cycle (staff briefing, mapping observations, data analysis and reporting, staff feedback, and action planning) that supports delivery of person-centered care. Two staff from the 31 intervention care homes were trained in DCM and asked to deliver 3 cycles over a 15-month period, supported by a DCM expert during cycle 1. Implementation data were collected after each mapping cycle. There was considerable variability in DCM implementation fidelity, dose, and reach. Not all homes trained 2 mappers on schedule, and some found it difficult to retain mappers. Only 26% of homes completed more than 1 cycle. Future DCM trials in care home settings should consider additional methods to support intervention completion including intervention delivery being conducted with ongoing external support.

## Introduction

Care homes provide care and support to up to 38% of people with dementia,^1,2^ with the majority of people living in care homes having dementia.^2,3^ Despite dementia being their core business, the quality of care home care for people with dementia is variable.^4,5^ Poor quality care for people with dementia is associated with an increase in behaviors such as agitation, apathy, and aggression.^6,7^ Although the need for psychosocial approaches to support good quality care is recognized,^8^ there are limited evidence-based interventions to support this^9,10^ and challenges in the widespread implementation of such interventions into everyday practice.^10,11^

Dementia Care Mapping™ (DCM)^12,13^ is a manualized, established intervention^14^ developed by the University of Bradford, United Kingdom, and used internationally in care home settings.^15^ It is an observational and practice development tool, implemented as quality improvement cycles, which aim to support the delivery of person-centered dementia care.^16^ Standard implementation is led by care home staff who attend a 4-day training program in use of DCM. The process includes 5 components: briefing staff about DCM, care practice observation using standardized coding frames, data analysis and summary report production, feedback of findings to the staff team, and action planning for practice development at individual resident and care home levels.^17^ Cycles are repeated every 4 to 6 months as part of a care home’s ongoing quality improvement.^16^

Reported benefits of DCM implementation in care homes include reduced resident agitation, depression, anxiety, and neuropsychiatric symptoms and improved quality of life.^18–21^ Some DCM trials, however, have not found positive outcomes for residents compared to usual care control.^22,23^ DCM implementation has also been reported to improve the quality of staff–resident care interactions,^19^ reduce staff burnout,^24^ and improve staffs’ feelings about^23^ and their connection with residents.^18^ DCM trials have faced challenges with implementation at the system level,^22,23^ a challenge also highlighted in a recent systematic review of DCM implementation.^25^ Intervention implementation and fidelity (the extent to which core components are delivered as intended in the research protocol) is important to investigate, to support interpretation of trial outcomes.^26^ Few DCM studies have been conducted as randomized controlled trials, with only 2 reporting full implementation procedures and process evaluation results.^27,28^ Thus, relatively little is known about the particular DCM implementation strategies that have proved effective.^29^ A German study reported largely good adherence to delivery of the requisite number of DCM cycles and cycle components.^27^ However, wider staff engagement in feedback sessions was low in 2 of the 6 intervention homes, and staff were critical of the quality of DCM delivery. In a Dutch study, involving 13 care units across 5 nursing home sites, DCM intervention adherence was variable.^28^ Two care units undertook no cycles of DCM, and completion of all DCM cycles across the other care units was variable. The limited available evidence and varied DCM implementation conditions and adherence in trials to date make interpretation of results challenging. More widely, it raises questions about whether randomized controlled trials that have found psychosocial interventions for dementia to be ineffective and not cost-effective might be explained by poor implementation adherence.^30^

The DCM EPIC cluster randomized controlled trial aimed to assess the effectiveness and cost-effectiveness of DCM in care home settings, including a full process evaluation to understand implementation processes and issues. This article reports on the DCM intervention delivery. It aimed to answer the following questions:

1. How was DCM delivered, compared to protocol?
2. What DCM components were delivered (fidelity, dose, adaptions and reach) compared to protocol?

## Methods

### Design and Setting

The full trial details are reported in the protocol.^31,32^ In summary, the DCM EPIC trial was a pragmatic, multicenter, cluster randomized controlled trial of DCM plus usual care (intervention) versus usual care alone (control). Sites were residential and nursing homes that provided care for people with dementia in 3 regions of the United Kingdom. Fifty care homes were recruited with 31 randomized to intervention and 19 to control. Data were collected by blinded researchers at baseline (prerandomization) and 6- and 16-month follow-up. The Medical Research Council guidance on process evaluations was followed.^33^ This included assessing the implementation process in terms of how delivery was achieved and what was delivered, as measured by fidelity (delivery as intended), dose (quantity of delivery), adaptions (changes made by individual sites), and reach (whether the intended audience received the intervention).

### Intervention

DCM implementation was described in the study protocol following standard procedures reported in the DCM manual and guidance.^13,34^ Two staff members per home were selected by the home manager to train to use DCM (called mappers), using a set of “mapper qualities criteria.” Mappers completed a standard 4-day DCM Basic User course that consists of information on person-centered dementia care, use of the 4 DCM coding frames and their application rules, and instruction on completing the DCM cycle components including staff briefing, data analysis, report writing, staff feedback, and action planning. They were then requested to implement 3 DCM cycles, comprised of briefing, observation, data analysis and reporting, feedback, and action planning. Per cycle, mappers were asked to deliver the following standard DCM components: at least 1 formal briefing and 1 formal feedback session for staff (and additional informal sessions as required); observations on up to 5 residents per mapper; use all 4 of the DCM observational coding frames and make qualitative notes; observe for up to 6 hours over 1 or more days during a week; write reports summarizing data for the care home and each resident mapped including specific feedback points; and produce an action plan with at least 1 action point for each resident mapped and the whole home. “Mapper instruction packs” were provided in hard and electronic formats containing fidelity guidelines and standardized templates for data processing, action planning, and reporting.

While DCM EPIC was a pragmatic trial, aiming to investigate DCM’s effectiveness when implemented in a manner reflective of standard UK DCM use, a number of additional mechanisms were introduced to support consistent implementation, which could be feasibly introduced in usual practice. This included support for the first DCM cycle by an expert mapper from the research team. The expert mapper provided 2 days of desk-based support for preparation, data analysis, and report writing and spent 3 days in the care home to support briefing, mapping, feedback, and action planning. Other support mechanisms included telephone and e-mail support from a member of the trial team, sending SMS reminders, and a “mapping pack” of paperwork by post to mappers ahead of each cycle.

### Participants and Data Collection

All mappers were asked to return data on DCM component adherence and fidelity at the end of each mapping cycle. This included data collection forms to record the number and dates of briefing and feedback sessions and the number and role of staff members attending and deidentified copies of their DCM observation sheets, feedback report, and action plans. Expert mappers reported after cycle 1 on component completion, mapper skills, and any concerns regarding sustainability of DCM implementation in the home. One author (CS) and the trial manager completed a standard case review form for each home at each cycle that summarized implementation data.

### Data Analysis

Data were summarized using descriptive statistics in SAS software v9.4 or Stata v14.^35,36^

### Ethical Issues

All study participants gave formal written consent to participate. Ethical approval for the study was granted by NRES Committee Yorkshire and The Humber—Bradford Leeds (REC ref 13/YH/0016). The trial was registered with the International Standard Randomized Controlled Trial Register (ISRCTN) reference ISRCTN82288852.

## Results

The main trial outcomes are published elsewhere.^37^

### Data Return

There was variable compliance in return of DCM implementation documentation, despite a range of approaches by the trial team to increase return rates. These included sending multiple phone and e-mail remainders and unblinded researchers visiting some homes to collect documentation. Where documentation was returned, there were missing data on some intervention components. Where documentation was provided for a later DCM phase only (eg, mapping data or feedback report), we assumed that undocumented earlier phases (eg, briefing session) had occurred. In some cases, mappers verbally reported that a DCM cycle or components of it had been completed but did not provide any supporting documentary evidence. These were recorded as incomplete for trial purposes. Implementation of the DCM intervention was of interest owing to the pragmatic nature of the trial design. Due to satisfactory completion of DCM cycle 1, the difficulties in obtaining documentary evidence of DCM cycles 2 and 3 completion, and the incremental (phased) recruitment of care homes over 14-month period with a 16-month intervention period, early termination of the trial was not considered.

### Implementation Process: Mapper Training and Retention

Mapper training was delivered per protocol (within 2 months of randomization) in 21 (67.7%) of 31 homes. In 1 (3.2%) home, no mappers were trained. In 2 (6.5%) homes, only 1 mapper was trained. Retention of mappers was problematic. One or both mappers withdrew in 17 (54.8%) homes, with reasons including resignation, long-term sickness, maternity leave, and lack of management support to undertake mapping. At 16-month follow-up, 10 (32.3%) homes had no mappers in the role, 7 (22.6%) had 1 mapper, and 14 (45.2%) homes retained both mappers. Although there was funding to train additional mappers (eg, due to mapper resignation or sick leave), this was accessed in only 1 home. Reasons for not training additional mappers included insufficient time or a new mapper being unable to attend training before the trial’s end and being unable to identify a suitable replacement mapper.

### What Was Delivered? Mapping Cycles

**Figure 1.**
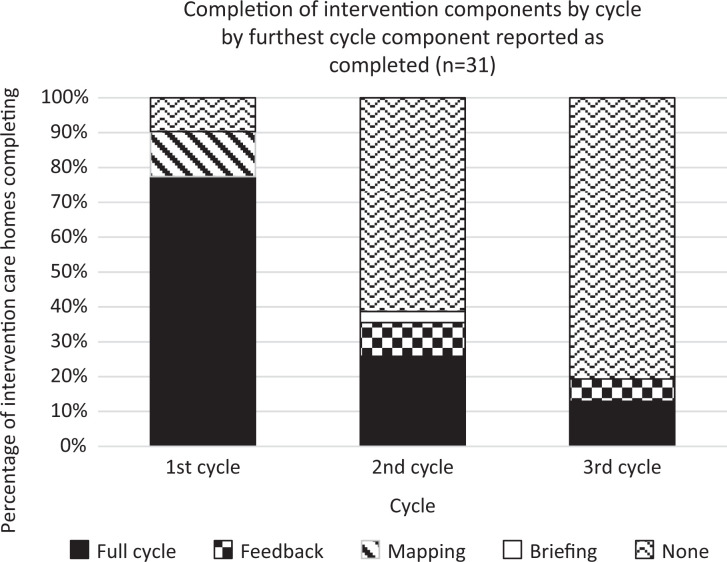
Completion of intervention components by cycle.

Implementation of the 3 DCM cycles across the 31 intervention homes was variable. Adherence is reported by cycle and highest level completed component in Figure 1. There was low adherence beyond the first supported cycle, with 16 (51.6%) homes only completing 1 cycle. Seven (22.6%) homes did not complete a full cycle, with 3 (9.7%) not completing any components, 4 (12.9%) homes completed 2 full cycles, and 4 (12.9%) completed 3 full cycles. Thus, the dose and fidelity of mapping cycles across the intervention homes was inconsistent, with only 4 (12.9%) homes achieving the per-protocol dose. The following sections examine fidelity, dose, adaptions, and reach per DCM component.

### Briefing

There was a substantial amount of missing data on briefing session completion (see Table 1). Where a briefing session was reported, the median number of staff receiving formal briefing increased per cycle from 10 in the first cycle to 20 in the third cycle. The majority of mappers also briefed staff informally. However, the range of numbers of staff briefed formally (3 to 28) and informally (2 to 31) in each home was wide across the 3 cycles. Therefore, in some homes, very few staff may have received DCM briefing. This indicates variable fidelity, dose, and reach of DCM briefing across the care homes.

**Table 1.** Summary of Briefing Session Fidelity in Homes Where Component Completed.

Summary of Briefing Sessions by Cycle			
	Cycle 1, n = 28	Cycle 2, n = 12	Cycle 3, n = 6
Number of formal briefing sessions held			
1	9 (32.1%)	7 (58.3%)	3 (50.0%)
2	4 (14.3%)	1 (8.3%)	1 (16.7%)
3	2 (7.1%)	1 (8.3%)	1 (16.7%)
Missing	13 (46.4%)	3 (25.0%)	1 (16.7%)
Total number of staff attended			
Mean (SD); missing	10.1 (4.52); 15	15.8 (7.44); 4	18.0 (8.19); 3
Median (range)	10 (3-20)	14.5 (8-28)	20 (9-25)
Informal briefing sessions held			
Yes	15 (53.6%)	10 (83.3%)	3 (50.0%)
No	1 (3.6%)	1 (8.3%)	2 (33.3%)
Missing	12 (42.9%)	1 (8.3%)	1 (16.7%)
Number of staff informally briefed			
Mean (SD); missing	10.5 (7.51); 14	13.1 (10.89); 4	19.3 (1.15); 3
Median (range)	8.5 (2.0-30.0)	7.0 (4.0-31.0)	20.0 (18.0-20.0)

Abbreviation: SD, standard deviation.

### Mapping Observations

Observation was conducted by 2 mappers for most cycles (see Table 2). The mean number of hours (7.8-9.4) and the median total number of residents observed per home (5-6) were reasonably consistent per cycle, although the range was not (4-12.4 hours and 2-10 residents), indicating variable observation dose and reach per cycle. Quality of DCM data coding improved over the cycles, based on rating of mappers’ use of all 4 DCM coding frames and making accompanying qualitative notes. While the percentage of mappers consistently achieving this remained similar at <50% per cycle, showing only moderate fidelity with the manualized DCM method, the proportion not meeting this criteria at all declined considerably over 3 cycles (from 35.7% to 16.7%).

**Table 2.** Summary of Mapping Observation Fidelity in Homes Where Component Completed.

Observation Adherence by Cycle			
	Cycle 1, n = 28	Cycle 2, n = 11	Cycle 3, n = 6
Number of mappers conducting observations			
1	1 (3.6%)	1 (9.1%)	1 (16.7%)
2	18 (64.3%)	10 (90.9%)	4 (66.7%)
Missing	9 (32.1%)	0	1 (16.7%)
Total mapping time, hours			
Mean (SD); missing	8.9 (2.76); 13	9.4 (2.30); 3	7.8 (0.43); 3
Median (range)	9.2 (4.0-12.4)	9.9 (6.5-12.3)	8.0 (7.3-8.0)
Total residents observed			
Mean (SD); missing	5.4 (1.79); 10	5.7 (2.41); 0	5.2 (1.79); 1
Median (range)	5 (2-8)	6 (2-10)	4 (4-8)
Used all 4 coding frames and made at least minimal qualitative notes			
Yes	9 (32.1%)	5 (45.5%)	2 (33.3%)
Partially	9 (32.1%)	6 (54.5%)	3 (50.0%)
No	10 (35.7%)	0	1 (16.7%)

Abbreviation: SD, standard deviation.

### Feedback

There was considerable missing data on delivery of feedback sessions and numbers of staff attending (Table 3). In each cycle, the majority (50%-73%) of homes documented delivery of a formal feedback session. While the median number of staff attending formal feedback increased per cycle, there was considerable variability, particularly during cycle 1 (2-17 staff attending). Likewise, there was sizeable variation across care homes on how many home- and resident-level feedback points were included in the report. This indicates substantial variability in DCM fidelity, dose, and reach of DCM feedback within and across the homes.

**Table 3.** Summary of Feedback Session Fidelity in Homes Where Component Completed.

Summary of Feedback Sessions by Cycle			
	Cycle 1, n = 24	Cycle 2, n = 11	Cycle 3, n = 6
Number of mappers participating in the feedback process			
1	1 (4.2%)	2 (18.2%)	0
2	13 (54.2%)	7 (63.6%)	3 (50.0%)
Missing	10 (41.7%)	2 (18.2%)	3 (50.0%)
Formal feedback sessions held N (%) Missing			
Yes	12 (50.0%)	8 (72.7%)	3 (50.0%)
No	2 (8.3%)	2 (18.2%)	1 (16.7%)
Missing	10 (41.7%)	1 (9.1%)	2 (33.3%)
Total number of formal feedback sessions			
Mean (SD); missing	1.8 (0.83); 12	1.4 (0.79); 4	1.0 (0.00); 3
Median (range)	2 (1-3)	1 (1-3)	1 (1-1)
Total number of staff attended formal feedback sessions			
Mean (SD); missing	9.6 (4.56) 12	12.3 (4.46) 5	12.3 (4.51) 3
Median (range)	9.0 (2-17)	11.5 (7-18)	12.0 (8-17)
N of care home feedback points			
Mean (SD); missing	5.0 (3.06); 14	3.7 (1.21); 5	6.0 (5.72); 2
Median (range)	4.5 (2-13)	3 (3-6)	5.5 (0-13)
Total number of mapped residents with feedback points			
Mean (SD); missing	4.4 (1.78); 12	4.2 (2.23); 5	3.5 (1.73); 2
Median (range)	4.5 (1-7)	5 (1-6)	4 (1-5)
Mean number of resident feedback points			
Mean (SD); missing	3.2 (2.12); 13	2.5 (0.93); 5	2.3 (0.96); 2
Median (range)	2.8 (0.8-7.8)	2.9 (1.0-3.3)	2.4 (1.3-3.3)

Abbreviation: SD, standard deviation.

### Action Planning

Of the homes that provided evidence of action planning, the percentage who produced a care home–level action plan increased per cycle (see Table 4), from just over 50% at cycle 1 to all homes in cycle 3, indicating higher fidelity of care home–level action planning in homes completing multiple DCM cycles. While the average number of care home action points produced per cycle was consistent, the range was wide, demonstrating dose variability across homes. Where homes commenced a DCM cycle but did not complete all components, action planning was the component most likely to be omitted. Resident action plans were received from 42% to 75% of homes per cycle, and 20% to 76% of residents observed had at least 1 action point written to support their care planning at each cycle, indicating poor protocol fidelity and inconsistent reach and dose of DCM action. Homes consistently used the trial’s action plan templates, indicating low adaptation of this component where completed.

**Table 4.** Summary of Action Planning Fidelity in Homes Where Component Completed.

Action Planning by Cycle			
	Cycle 1, n = 24	Cycle 2, n = 8	Cycle 3, n = 4
Care home action plan received, n (%)			
Yes	13 (54.2%)	6 (75.0%)	4 (100.0%)
No	6 (25.0%)	2 (25.0%)	0
Missing^a^	5	0	0
Number of care home action points			
Mean (SD)	4.9 (3.20)	5.2 (4.83)	5.0 (2.16)
Median (range)	4 (2-14)	3 (3-15)	4.5 (3-8)
Standard care home action plan template used			
Yes	13 (100.0%)	6 (100.0%)	3 (75.0%)
At least one resident action plan received, n (%)			
Yes	13 (41.9%)	6 (75.0%)	3 (75.0%)
No	6 (19.4%)	2 (25.0%)	1 (25.0%)
Missing^a^	5	0	0
Total number of residents with action points			
Mean (SD)	5.5 (1.85)	5.8 (2.86)	4.7 (1.15)
Median (range)	5 (3-8)	5.5 (2-10)	4 (4-6)
Mean number of action points per resident where plan completed			
Mean (SD); missing	2.0 (1.95)	2.0 (1.24)	1.8 (1.77)
Median (range)	1.6 (0.1-7.8)	2.2 (0.1-3.3)	1.3 (0.3-3.8)
Standard resident action plan template used where plan completed, n (%)			
Yes	12 (92.3%)	6 (75.0%)	2 (75.0%)
At least one action point per observed resident where plans completed			
Yes	5 (20.1%)	4 (66.7%) 25	1 (33.3%)
No	8 (33.3%)	2 (33.3%)	2 (66.7%)

Abbreviation: SD, standard deviation.

^a^Data may be missing in cycle 1 since components completed could be recorded via confirmation through data collection form completed by expert mapper, even if no mapping documentation received from the mappers.

## Discussion

When implementing a multicomponent care improvement intervention such as DCM, it is important to understand whether core components have been implemented as intended^26^ and to assess any implementation challenges associated with the different components.^38^ The results of this process evaluation show that across the 31 intervention homes, there was poor consistency in the fidelity, dose, and reach of DCM. This applied to the number of DCM cycles completed and to execution of each DCM intervention component per cycle.

Overall, there was low intervention fidelity and dose of DCM compared to trial protocol, and where cycles did occur, implementation quality was often low. These findings mirror those reported in other studies utilizing care home staff-led DCM cycles,^22,28^ and in other studies of complex interventions in long-term care settings where staff-led intervention implementation has been utilized.^39^ Reported barriers and facilitators to the trial’s DCM implementation, gained through qualitative interviews with mappers, managers, care home staff, and expert mappers, are discussed in full in a separate article.^40^ However, in summary, barriers and facilitators were evident at 4 levels, the individual mapper level, care home level, intervention level, and trial level. Care home–level barriers included low staffing levels, high staff turnover, lack of time to undertake DCM, and competing priorities. If the care home manager was not fully supportive of DCM, this was an identified barrier to DCM. In addition, external priorities took precedence such as regulatory inspections and any requirements they might place on a home. These contextual factors offer an explanation for why some homes failed to implement any full cycles of DCM and why so few homes did not conduct more than their first supported cycle.

Fidelity was weak for some components of DCM observation. While on average the length of observation and numbers of residents observed during each cycle adhered to the trial protocol, this was inconsistent when examining ranges. Of note was the relatively unchanging proportion of mappers who used all 4 DCM coding frames and who made qualitative notes during observations. Failure to consistently record DCM data in line with the manualized method creates doubts about the accuracy of the DCM data informing practice development. Interviews with mappers about the barriers and facilitators to DCM implementation indicated that there were DCM intervention-level barriers that included perceptions of DCM being too complex and time consuming, including the coding frames used during observations.^40^ Managers in the same study discussed how identifying staff with the requisite academic skills to be able to successfully implement DCM could be challenging within a care home environment. While senior staff might be more experienced and potentially able to use DCM, they were noted to be less likely to be able to be released to undertake DCM training and implementation, particularly in smaller nursing homes and in residential homes where there were fewer/no nurses.

The variability seen in availability and numbers of staff attending briefing and feedback sessions indicates low fidelity and reach of these core DCM components. Briefing sessions facilitate engagement of staff in the DCM process, and feedback sessions offer the crucial opportunity for staff to discuss the DCM findings, analyze their meaning and implications, and undertake action planning. These components are fundamental to staff ownership of practice improvement. Low staff engagement with DCM was also reported in a previous process evaluation study.^27^ When implementing complex interventions such as DCM in care home settings, engagement of the wider staff team^41,42^ and good communication around implementation^43,44^ are identified facilitators of adoption in practice. These factors were unlikely in homes where few staff were involved in DCM briefing or feedback. A range of care home–, mapper-, and intervention-level barriers and facilitators to staff engagement with DCM were identified in the process evaluation interview data from this study.^40^ These included the culture within the care home and whether staff were open to change, whether the care home manager supported the mappers in facilitating staff attendance at briefing and feedback sessions, whether the mappers were respected by and could easily engage their colleagues, and how well mappers were able to explain DCM and its potential benefits to others. This indicates that the readiness of the care home for DCM ahead of implementation as well as choosing individuals with the requisite skills and status within the home to lead DCM are important prerequisites that may serve to support or undermine the likelihood of engaging the full staff team with the DCM process.

There was also considerable variability in the execution of DCM action planning across the intervention homes, with reported inconsistency in reach and dose. This included whether care home– and individual resident-level action plans were produced and how many action points were written per plan. It is possible that action points were identified during DCM feedback sessions but that formal action plans were not produced. However, a lack of formal written records of practice development plans and only small numbers of staff attending formal feedback sessions in some of the care homes mean it is unlikely all staff were exposed to verbally produced action plans. These protocol lapses potentially jeopardized DCM-related practice change and limited opportunities for monitoring development over time. The interview data from this process evaluation indicated that the mappers found the skills required to undertake data analysis, report writing, and the development of action plans consistently challenging.^40^ They frequently reported not having the IT skills to produce the standardized feedback reports, and due to this, the process took much longer than anticipated. Mappers were also required to use skills they had not previously had to employ, such as engaging colleagues in discussion of practice development issues and accurate written recording of outcomes. This was compounded by low literacy and numeracy skills and use of English as a second or additional language by some mappers. Given action plans require completion of further paperwork, their inconsistent completion may therefore be related to fatigue, competency, and the amount of time required to complete paperwork. The amounts of paperwork required during the DCM process were identified as a major barrier to its use by mappers.

In a German process evaluation,^27^ they concluded that the well-defined, prestructured components of DCM could be easily implemented by care home staff, with biggest barrier to implementation being the translation of action plans into practice change. In contrast to these findings, and in line with those of van der Ven et al,^28^ our study indicates that most care home staff were unable to deliver the standard, manualized components of DCM with sufficient fidelity or dose. DCM is identified by mappers as a complex tool hampering their ability to use it accurately.^25,45^ The complexity of an intervention is a potential barrier to implementation identified in other care home trials.^44,46,47^ The interview data from this process evaluation^40^ indicated that not only was the complexity of DCM as a tool a barrier to implementation, but mappers often lacked the skills and confidence to lead a process of change within the care home. This was further hampered by setting conditions within the environment which could serve to facilitate or undermine DCM, including the culture, management support for DCM, and staff willingness to engage with practice change.

In this study, loss of mappers was a common occurrence, with one or both mappers withdrawing in 54.8% of homes by 16-month follow-up due to a range of staff and contextual issues, including lack of managerial support to map. A further challenge was the limited opportunity to identify and train further mappers with the requisite skills within the trial period. High staff turnover rates within care home settings and the consequent need to facilitate regular staff retraining have been identified as a challenge to implementing and sustaining complex interventions.^43,44,48,49^ Since the majority of completed DCM cycles were undertaken by 2 mappers, the loss of 1 mapper in a care home is likely to jeopardize continued use of DCM. Thus, DCM implementation is highly vulnerable to staffing-related issues. The process evaluation interview data supported this, with lone mappers indicating that undertaking a DCM cycle alone felt complex, time consuming, and overwhelming.^40^

Overall, this process evaluation has identified a range of challenges to the accurate and sustained implementation of DCM by care home staff. These findings also have relevance to the use of other complex interventions within care home settings. Challenges with return of data on DCM cycle completion were a major barrier to intervention fidelity monitoring during the trial. The reliance on care home mappers to return these data and the inconsistency between verbal and written reports of cycle completion suggest researchers conducting care home trials need to consider if and how they will collect fidelity data. Mechanisms that do not rely on care home staff to return fidelity data should be considered and may be preferable.

While mapper selection criteria were utilized in this trial to assist manager to select appropriate individuals for the role, the fidelity data suggest that this may not have led to selection of the right individuals with the requisite skills to accurately implement and sustain use of DCM. Interviews with managers^40^ indicated that the pool of suitable staff who met the mapper selection criteria may be limited. Experienced staff with the requisite skill were usually working in senior roles and it was more difficult to release them to attend training and complete mapping. Completion of the standard 4-day DCM Basic User training course did not equip all mappers to use DCM accurately, even with support from an external expert. Replacement of mappers who left their post was therefore challenging, and many mappers lacked the confidence and skills to continue to implement it alone. Given any intervention designed to change care home practice is likely to require an initial high-level of skilled mapper leadership, future use of DCM or other complex interventions should consider if and how care home staff can be equipped to develop such skills and whether alternative models of implementation are required. For example, ongoing support from an external expert mapper who can continue to work alongside care home staff over the long term may be beneficial.

A range of care home–level factors such as management support and staff willingness to engage with change also impact DCM fidelity, reach, and dose. Given the pragmatic, explanatory nature of this trial, care homes were selected randomly from 3 geographic regions. This finding indicates that some care homes may not provide the right setting conditions for DCM and potentially for implementation of other complex interventions. Consideration may need to be given within an intervention as to how the care home culture can be made ready for implementation ahead of commencement.

Incentives for care homes to undertake intervention implementation is also a consideration. In this trial, care homes were not provided with funding to backfill staff to attend DCM training or to undertake DCM cycles. If this had been provided, more managers may have been supportive of giving mappers enough dedicated time to complete the DCM cycles. While this may have improved the implementation dose and sustainability, it is unlikely to have addressed the accuracy with which DCM was implemented or the challenges managers faced in identifying individuals with the requisite skills to lead DCM. The small number of care homes completing more than their first supported DCM cycle and variability in the fidelity and reach per component per cycle makes it difficult to draw any conclusions about fidelity between cycles.

### Limitations

The study had a number of limitations. Given the challenges we experienced in the return of implementation data from mappers and the degree of missing implementation data, the recorded compliance data may be subject to inaccuracies of both under- and overreporting. We were also unable to assess the quality of delivery of some components, for example, briefing and feedback sessions, where carried out. Therefore, reported completion of a DCM component is not an indicator of completion quality.

## Conclusions

In this pragmatic trial of DCM using standard care home staff-led cycles, the implementation process, fidelity, dose, and reach were found to vary across cycles and homes. Only 4 homes implemented DCM according to the trial protocol. DCM Basic User training did not prepare all care home mappers to implement DCM accurately and to sustain its use. There was variability in DCM reach related to both mapper implementation and wider care home–level issues. Identifying individuals with the requisite skills and time to implement DCM appear to be challenging in care home settings. Likewise, whether care homes have the right culture and ethos to successfully implement DCM warrants consideration ahead of commencing mapper training. This finding is informative, given the use of well-established DCM implementation procedures. Future complex intervention trials in care home settings will likely benefit from further research on suitable evidence-based implementation strategies in this setting. Consideration may need to be given to complex intervention delivery being conducted wholly through, or with ongoing support of, external translation experts.
